# LPS/TLR4 Signaling Enhances TGF-β Response Through Downregulating BAMBI During Prostatic Hyperplasia

**DOI:** 10.1038/srep27051

**Published:** 2016-05-31

**Authors:** Yao He, Zhenyu Ou, Xiang Chen, Xiongbing Zu, Longfei Liu, Yuan Li, Zhenzhen Cao, Minfeng Chen, Zhi Chen, Hequn Chen, Lin Qi, Long Wang

**Affiliations:** 1 Department of Urology, Xiangya Hospital, Central South University, Changsha, Hunan 410008, China; 2 Department of Gynecologic Oncology, Hunan Provincial Tumor Hospital and Affiliated Tumor Hospital of Xiangya Medical School, Central South University, Changsha, Hunan 410013, China

## Abstract

Compelling evidence suggests that benign prostatic hyperplasia (BPH) development involves accumulation of mesenchymal-like cells derived from the prostatic epithelium by epithelial-mesenchymal transition (EMT). Transforming growth factor (TGF)-β induces EMT phenotypes with low E-cadherin and high vimentin expression in prostatic epithelial cells. Here we report that LPS/TLR4 signalling induces down-regulation of the bone morphogenic protein and activin membrane-bound inhibitor (BAMBI), which enhances TGF-β signalling in the EMT process during prostatic hyperplasia. Additionally, we found that the mean TLR4 staining score was significantly higher in BPH tissues with inflammation compared with BPH tissues without inflammation (5.13 ± 1.21 and 2.96 ± 0.73, respectively; *P* < 0.001). Moreover, patients with inflammatory infiltrate were more likely to have a higher age (*P* = 0.020), BMI (*P* = 0.026), prostate volume (*P* = 0.024), total IPSS score (*P* = 0.009) and IPSS-S (*P* < 0.001). Pearson’s correlation coefficient and multiple regression analyses demonstrated that TLR4 mRNA expression level was significantly positively associated with age, BMI, serum PSA levels, urgency and nocturia subscores of IPSS in the inflammatory group. These findings provide new insights into the TLR4-amplified EMT process and the association between TLR4 levels and storage LUTS, suggesting chronic inflammation as vital to the pathogenesis of BPH.

Benign prostatic hyperplasia (BPH) is the nonmalignant overgrowth of the prostatic tissue within the transition zone^1^. Despite its high prevalence, the exact aetiology of BPH remains unresolved^2^,^3^. Several different partially overlapping theories, involving hormones, inflammatory mediators, embryonic reawakening and epithelial-stromal interactions, have been proposed, but each seems to be operative to some extent and there is no consensus as to which is the primary one. Basic science and clinical studies have recently suggested that BPH might be an immune-mediated inflammatory disease, whereby antigenic stimuli may lead to chronic inflammatory reactions within the prostate that result in prostatic tissue rebuilding and overgrowth^4^,^5^.

Prostate epithelial cells secrete inflammatory cytokines that lead to the activation of signalling pathways that regulate cell proliferation, apoptosis and differentiation^6^,^7^. Stimulation by lipopolysaccharide (LPS), a major component of the outer membrane of Gram-negative bacteria, can lead to local infections or inflammatory processes through the Toll-like receptor 4 (TLR4)-mediated signalling pathway^8^,^9^. TLR4, a receptor for LPS, is one of the strongest inducers of inflammation^10^. Recent studies have suggested that LPS/TLR4 signalling plays a key role in the process of chronic inflammation inducing organ fibrogenesis and cancer development^11^,^12^,^13^. However, to our knowledge, there are few reports concerning the TLR4-mediated signalling pathway in prostatic epithelial cells. Thus, we hypothesised that the TLR4-mediated signalling pathway may be involved in the development of BPH and its molecular mechanisms.

Recent evidence suggests that BPH development involves accumulation of mesenchymal-like cells derived from the prostatic epithelium by epithelial-mesenchymal transition (EMT)^14^,^15^,^16^. Transforming growth factor-β1 (TGF-β1) is a pleiotropic cytokine that induces remodelling of epithelial cells and is a strong extracellular inducer of EMT in various normal and cancer cell types^17^,^18^,^19^. Pei *et al.*^20^ found that the expression and secretion of TGF-β1 were upregulated after LPS treatment. Kim *et al.*^21^ injected LPS into rat prostates to mimic prostatitis and observed profound EMT features in LPS-treated rat prostates. Thus, we hypothesised that there might be an interaction between LPS/TLR4 and TGF-β signalling pathways.

The pseudoreceptor BAMBI (bone morphogenic protein and activin membrane-bound inhibitor) is a member of the TGF-β type I receptor family that lacks an intracellular kinase domain and functions as an inhibitor of the TGF-β signalling pathway^22^,^23^. Degen *et al.*^24^ reported that BAMBI mRNA is highly expressed in human placenta, spleen and kidney medulla, low in liver, prostate, gut and kidney cortex and absent in muscle and lung. However, no report has examined BAMBI expression in BPH tissues.

In this study, we report that BAMBI acts as a regulator between LPS/TLR4 signalling and TGF-β-induced EMT *in vitro* during prostatic hyperplasia. We explored a hypothetical molecular mechanism underlying inflammatory BPH, namely that TLR4-dependent downregulation of BAMBI might be an important mechanism of TLR4 activation intensifying TGF-β-mediated EMT. In addition, we investigated the expression of TLR4, BAMBI, p-Smad2/3 and clinical findings in prostate tissues with or without inflammatory infiltration. We further examined the correlation between TLR4 mRNA expression and clinical findings, including the severity of lower urinary tract symptoms (LUTS), in the prostate of BPH patients with inflammatory infiltrate.

## Results

### LPS synergize with TGF-β1-induction of EMT in BPH-1 cells

**Figure 1:**
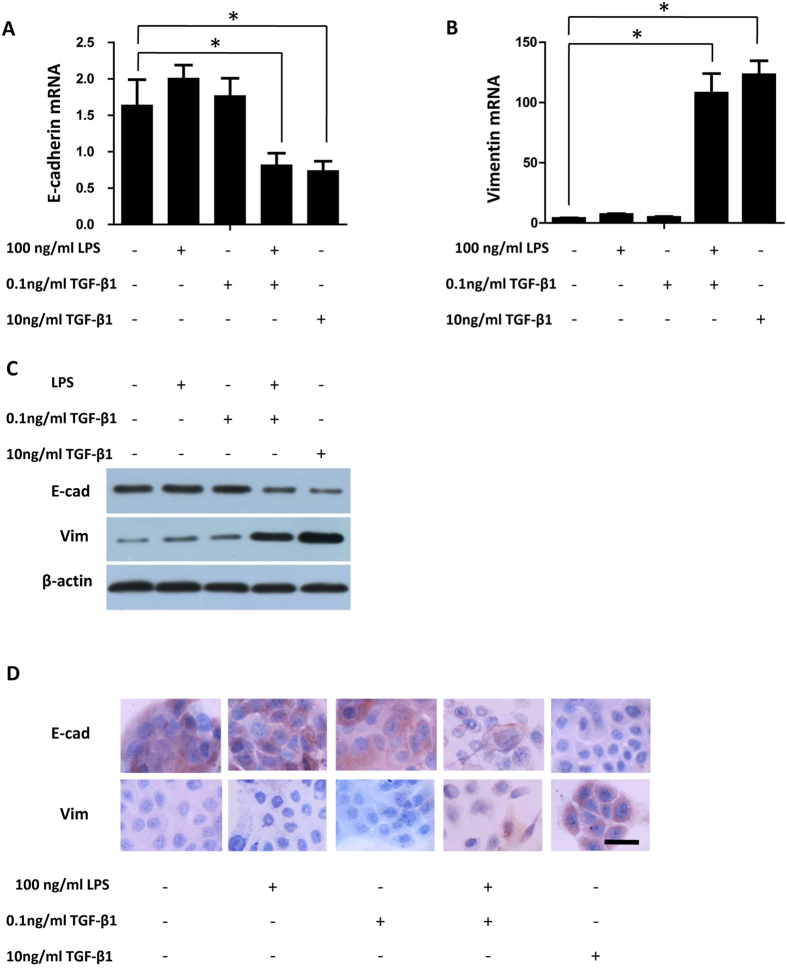
LPS in the presence of TGF-β1 induces changes in expression of EMT-associated markers in BPH-1 cells. BPH-1 cells were incubated with LPS (100 ng/ml) in the presence or absence of TGF-β1 (0.1 or 10 ng/ml). (**A,B**) E-Cadherin and vimentin mRNA expression were examined by qPCR. (**C**) Western blot analysis of E-cadherin and vimentin. Values shown indicate relative levels corrected for β-actin. (**D**) Immunocytochemical staining of E-cadherin and vimentin in BPH-1 cells after treatment with LPS in the presence or absence of TGF-β1 (0.1 or 10 ng/ml). Scale bar, 50 μm. *P < 0.05, determined by multifactorial ANOVA.

To examine whether the LPS can modulate the TGF-β1 signalling pathway in stimulated BPH-1 cells, cells were incubated with LPS in the presence or absence of 0.1 ng/ml TGF-β1. Based on the literature, we used 10 ng/ml TGF-β1 as the positive control and 0.1 ng/ml TGF-β1 as the negative control. Western blot and qPCR analysis showed that LPS 100 ng/ml or 0.1 ng/ml TGF-β1 alone had no significant effect on E-cadherin or vimentin mRNA and protein expression. However, 10 ng/ml TGF-β1 resulted in a marked reduction in E-cadherin mRNA and protein expression and increase in vimentin mRNA and protein levels. Furthermore, although less obvious than that of 10 ng/ml TGF-β1, LPS in the presence of 0.1 ng/ml TGF-β1 also resulted in decreased E-cadherin mRNA and protein expression and increased vimentin mRNA and protein levels (Fig. 1A–C), implicating a role for LPS in sensitising BPH-1 cells to the effects of exogenous TGF-β1. We found similar results in immunocytochemistry analysis (Fig. 1D).

### LPS/TLR4 axis affects Smad-mediated canonical TGF-β signalling pathway to drive EMT

**Figure 2:**
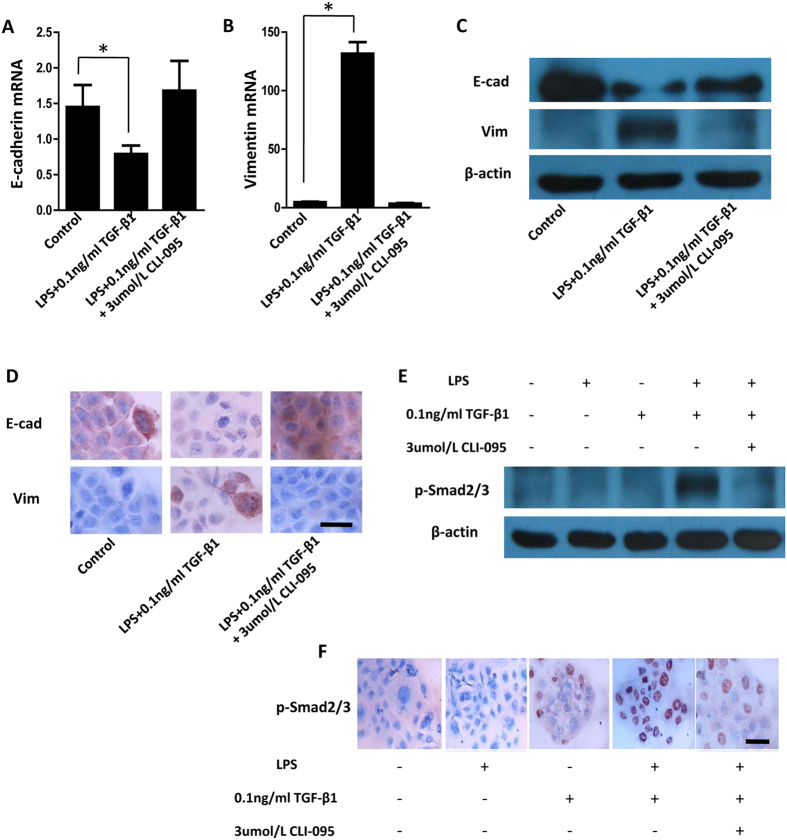
LPS/TLR4 axis affects Smad-mediated canonical TGF-β signalling pathway to drive EMT. BPH-1 cells were incubated with LPS (100 ng/ml) and TGF-β1 (0.1 ng/ml) in the absence or presence of the TLR4 inhibitor CLI-095 (3 μmol/L). (**A,B**) Total RNA was subjected to qPCR. (**C**) Whole cell lysates were examined for E-cadherin and vimentin protein levels by western blot analysis. Values shown indicate relative levels corrected for β-actin. (**D**) BPH-1 cells were fixed, incubated with LPS and TGF-β1 (0.1 ng/ml) in the absence or presence of CLI-095 (3 μmol/L), and examined by immunocytochemical staining. Scale bar, 50 μm. (**E**) BPH-1 cells were incubated with LPS and TGF-β1 (0.1 ng/ml) in the absence or presence of CLI-095. Whole cell lysates were examined by using western blot analysis for p-Smad2/3 protein levels. (**F**) BPH-1 cells were fixed, incubated with LPS and TGF-β1 (0.1 ng/ml) in the absence or presence of CLI-095, and examined by immunocytochemical staining. Scale bar, 50 μm. *P < 0.05, determined by multifactorial ANOVA.

As TLR4 is a critical receptor that transduces the LPS signal, we investigated the role of the LPS/TLR4 axis in mediating the TGF-β1-sensitising effects of LPS in BPH-1 cells. We used the potent TLR4 inhibitor CLI-095, which blocks TLR4 signalling pathways by binding to the intracellular domain of TLR4 and blocking downstream signalling events. qPCR and immunoblotting demonstrated that the synergistic effect of LPS and TGF-β1 (0.1 ng/ml) in decreasing E-cadherin mRNA and protein expression was abrogated by CLI-095 (Fig. 2A,C). Furthermore, the upregulation of vimentin mRNA and protein levels induced by combined LPS and TGF-β1 (0.1 ng/ml) was also completely blocked by CLI-095 (Fig. 2B,C). Immunocytochemical showed similar results (Fig. 2D).

Smad signalling plays important regulatory functions in TGF-β-mediated EMT. Therefore, we next analysed the role of Smad signalling. Western blot analysis showed that although LPS or TGF-β1 (0.1 ng/ml) treatment alone had no apparent effect on Smad2/3 phosphorylation, the combined LPS and TGF-β1 (0.1 ng/ml) treatment strongly induced a upregulation of p-Smad2/3 protein levels, and this effect was completely abrogated by CLI-095 pretreatment (Fig. 2E). Immunocytochemistry revealed no marked changes in the intensity of p-Smad2/3 staining in LPS-treated BPH-1 cells compared with control cells, with only a few nuclei positive for p-Smad2/3 after TGF-β1 (0.1 ng/ml) treatment (Fig. 2F). However, most nuclei showed positive p-Smad2/3 expression after combined treatment of LPS and TGF-β1 (0.1 ng/ml). Moreover, this effect was markedly suppressed by CLI-095.

### Functional activation of LPS/TLR4 axis mediates BAMBI downregulation

**Figure 3:**
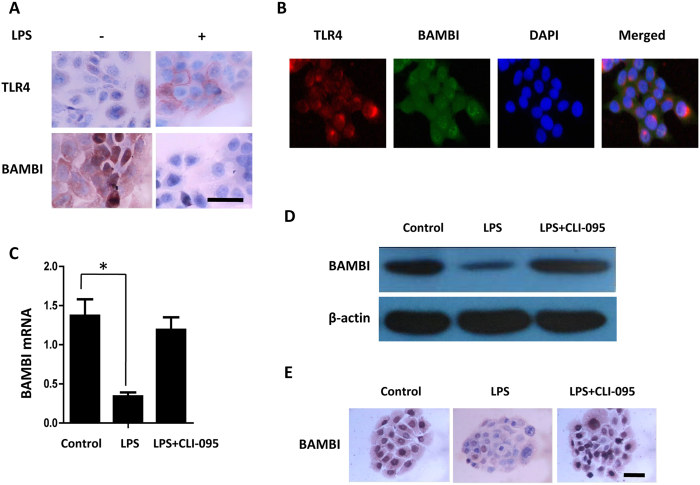
Functional activation of the LPS/TLR4 axis mediates BAMBI downregulation. (**A**) Immunocytochemical analysis was used to assess the expression of TLR4 and BAMBI in untreated and 100 ng/ml LPS-treated BPH-1 cells. Scale bar, 50 μm. (**B**) Double immunofluorescent technique was performed to examine the localisation of TLR4 and BAMBI in BPH-1 cells. TLR4 was localised in the cell membrane and cytosol (red) and BAMBI was detected in the membrane, cytoplasm and some nuclei (green). Scale bar, 50 μm. (**C**) BPH-1 cells were incubated with LPS (100 ng/ml) or pretreated with CLI-095 (3 μmol/L). Total RNA was subjected to qPCR. (**D**) Western blotting analysis of expression of BAMBI in control and LPS alone or pretreated with CLI-095 (3 μmol/L) for 1 h treated BPH-1 cells. (**E**) Immunocytochemical analysis of expression and localisation of BAMBI (brown) after LPS treatment or pretreated with CLI-095. Scale bar, 50 μm. *P < 0.05, determined by multifactorial ANOVA.

To investigate the mechanism of TLR4-mediated enhancement of TGF-β/Smad signalling, we focused on BAMBI, which functions as a general negative regulator of TGF-β-Smad signalling. We first examined the expression of TLR4 and BAMBI in BPH-1 cells using immunocytochemistry. TLR4 is expressed in the membrane and cytoplasm of BPH-1 cells and up-regulated after LPS treatment. BAMBI is expressed in the membrane, cytoplasm and some nuclei of BPH-1 cells and is markedly down-regulated after LPS treatment (Fig. 3A). Double staining of TLR4 and BAMBI in BPH-1 cells revealed that TLR4 staining was observed in the cell membrane and cytosol of BPH-1 cells and colocalised with BAMBI (Fig. 3B). Next, we investigated whether the LPS/TLR4 axis mediates BAMBI downregulation. Real-time PCR results showed that LPS treatment alone induced a significant downregulation (by three-fold) of BAMBI mRNA expression in BPH-1 cells compared with control cells. However, when BPH-1 cells were pretreated with CLI-095, the downregulation of BAMBI was abrogated (Fig. 3C). A similar tendency was observed in BPH-1 cells using western blot and immunocytochemistry (Fig. 3D,E).

### TLR4-mediated BAMBI downregulation enhances the intensity of TGF-β-induced EMT responses

**Figure 4:**
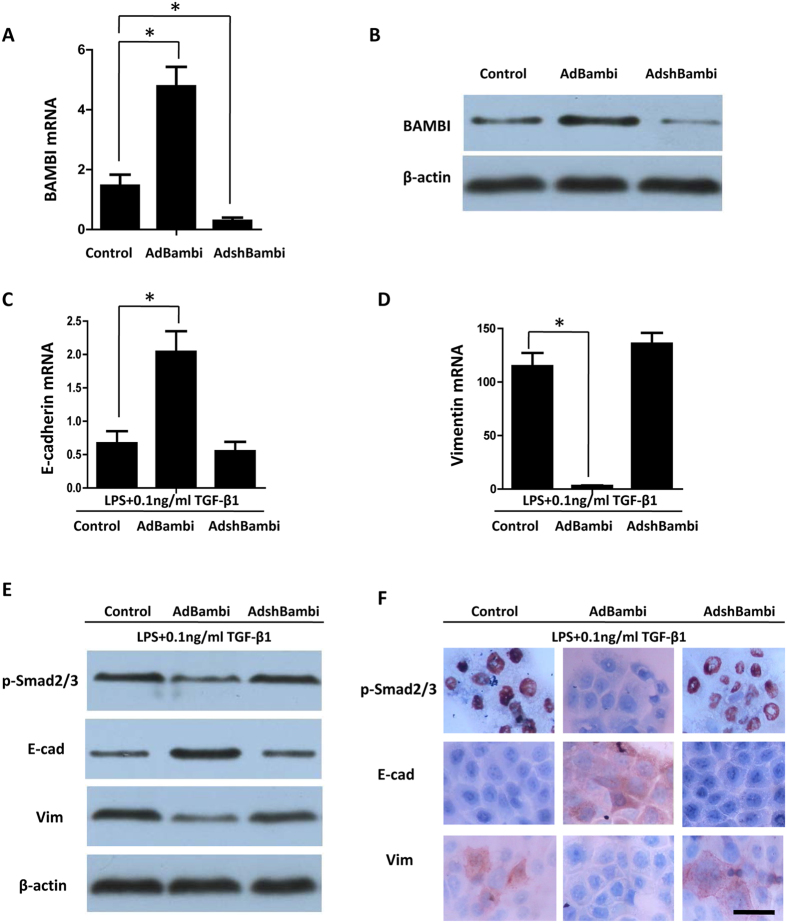
TLR4-dependent downregulation of BAMBI enhances the intensity of TGF-β-mediated EMT responses. (**A,B**) BPH-1 cells were infected with adenoviruses expressing AdBambi or AdshBambi. qPCR and Western blot were used to confirm successful infection of the adenoviruses. (**C,D**) BPH-1 cells were pretreated with LPS (100 ng/ml) for 24 h and then treated with TGF-β1 (0.1 ng/ml) for an additional 48 h. Total RNA was subjected to qPCR. (**E**) Whole cell lysates were examined for p-Smad2/3, E-cadherin, and vimentin protein levels using western blot analysis. (**F**) Immunostaining of p-Smad2/3, E-cadherin, and vimentin. Scale bar, 50 μm. *P < 0.05, determined by multifactorial ANOVA.

To test the hypothesis that TLR4-dependent downregulation of BAMBI might be an important mechanism of TLR4 activation intensifying TGF-β-mediated EMT, we infected BPH-1 cells with BAMBI adenovirus (AdBambi) or BAMBI shRNA (AdshBambi) and used the untransfected BPH-1 cells as the blank control. We first examined mRNA and protein expression of BAMBI in cells by qPCR and western blot and confirmed successful infection of adenoviruses in AdBambi-infected and AdshBambi-infected cells (Fig. 4A,B).

We next measured the expression of p-Smad2/3, E-cadherin, and vimentin after stimulation of infected cells with LPS (100 ng/ml) and TGF-β1 (0.1 ng/ml). We found significantly lower levels of p-Smad2/3 in AdBambi-infected BPH-1 cells than in AdshBambi-infected and uninfected BPH-1 cells after LPS plus TGF-β1 (0.1 ng/ml) treatment (Fig. 4E). There was no significant difference in p-Smad2/3 expression between AdshBambi-infected and uninfected BPH-1 cells. These results were supported by immunocytochemistry. P-Smad2/3 was highly expressed in the nuclei of both uninfected and AdshBambi-infected BPH-1 cells after LPS plus TGF-β1 (0.1 ng/ml) treatment, whereas the AdBambi-infected BPH-1 cells were almost negative for p-Smad2/3 (Fig. 4F).

We found a three-fold to four-fold upregulation of E-cadherin mRNA expression in AdBambi-infected BPH-1 cells compared with AdshBambi-infected and uninfected BPH-1 cells after LPS plus TGF-β1 (0.1 ng/ml) treatment (*P* < 0.05) (Fig. 4C). We also observed a significant decrease of vimentin mRNA expression in AdBambi-infected BPH-1 cells compared with control or AdshBambi-infected BPH-1 cells (*P* < 0.05) (Fig. 4D). No statistically significant difference was observed in AdshBambi-infected and uninfected BPH-1 cells for E-cadherin and vimentin mRNA expression. A similar trend was observed in western blot and immunocytochemistry (Fig. 4E,F).

### TLR4 and BAMBI expression differences in prostate tissues with or without inflammatory infiltration

**Figure 5:**
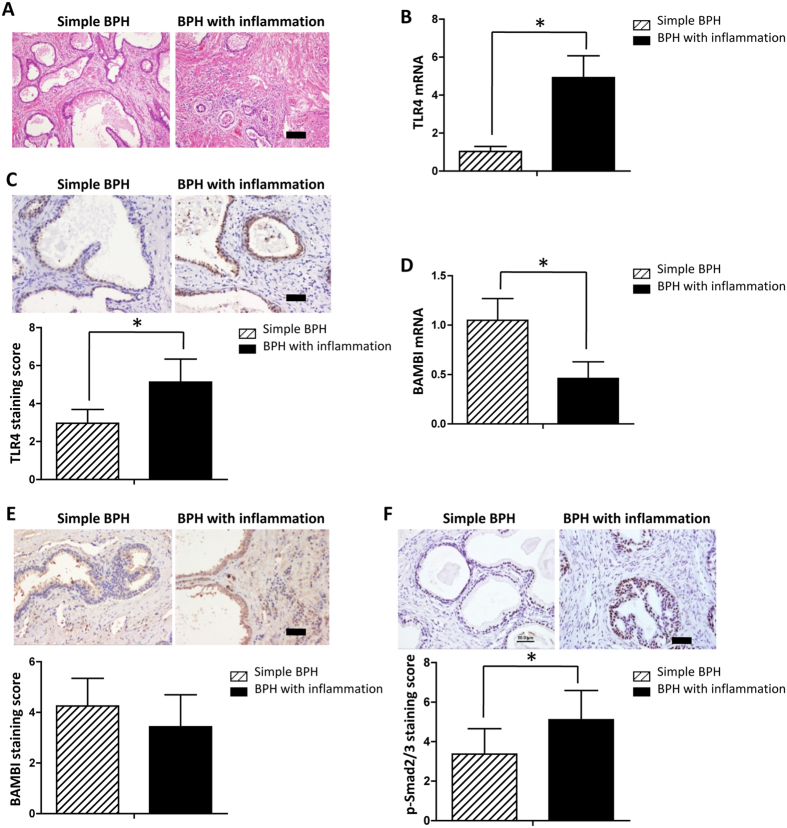
Expression of TLR4, BAMBI, p-Smad2/3 in BPH patient samples. Prostate specimens obtained by TURP from patients with BPH (n = 62) were stained by H&E. (**A**) A total of 47 of 62 patients had inflammatory cells infiltrating BPH tissues, while 15 of 62 patients had no obvious inflammatory cellular infiltration in BPH tissues. Scale bar, 100 μm. (**B**) Prostatic levels of TLR4 mRNA were measured by qPCR in the inflammatory group (n = 47) and non-inflammatory group (n = 15). *P < 0.001. (**C**) Immunostaining of TLR4. TLR4 (brown staining) was expressed in the cytoplasm in the glandular epithelium cells. The mean TLR4 staining score in the inflammatory group was compared with the non-inflammatory group. Scale bar, 50 μm. *P < 0.001. (**D**) Prostatic levels of BAMBI mRNA were measured by qPCR in the inflammatory group (n = 47) and non-inflammatory group (n = 15). *P < 0.001. (**E**) BAMBI (brown staining) was expressed in the cytoplasm and several nuclei of glandular epithelium cells and some stromal cells. The mean BAMBI staining score in the inflammatory group was compared with non-inflammatory group. Scale bar, 50 μm. *P* = 0.095. (**F**) P-Smad2/3 was expressed in the nuclei of glandular epithelial cells but not in the basal cells. The mean p-Smad2/3 staining score in the inflammatory group was compared with non-inflammatory group. Scale bar, 50 μm. *P < 0.001, determined by Wilcoxon rank sum test.

To further verify our *in vitro* findings, we analysed TLR4 and BAMBI expression in human prostate tissues. Patients characteristics and statistics are presented in Table 1. Overall, 47 of the 62 patients (76%) showed inflammatory infiltration within the prostate tissue samples, and the remaining 15 patients (24%) did not present pathologic characteristics suggestive of inflammation response (Fig. 5A). There was a significantly different distribution in age and BMI between the two groups. Compared with patients without inflammatory status, patients with inflammatory infiltrate were more likely to have a higher age (*P* = 0.020), BMI (*P* = 0.026), prostate volume (*P* = 0.024), total IPSS score (*P* = 0.009) and IPSS-S (*P* < 0.001); however, no significant difference was noted in IPSS-V. As shown in Table 1, a significantly higher frequency (*P* = 0.008), urgency (*P* = 0.011) and nocturia (*P* = 0.036) subscores of IPSS were found in patients with inflammatory status compared with patients without inflammatory infiltrate. No significant differences for intermittency, frequency, incomplete emptying and weak stream subscores of IPSS were observed among patients with and without inflammatory status. No significant difference was found between the two groups in prostate-specific antigen (PSA), quality of life (QOL) index, maximum flow rate (Qmax), and postvoid residual (PVR).

Additionally, we observed that the mean TLR4 mRNA expression level was higher in the inflammatory group compared with the non-inflammatory group, and the difference was statistically significant (Fig. 5B) (*P* < 0.001). Immunohistochemistry demonstrated that in contrast to non-inflammatory BPH tissues with low TLR4, inflammatory BPH tissues expressed higher levels of cytoplasmic TLR4 in a significant proportion of luminal epithelial cells. The mean TLR4 staining score was significantly higher in tissues with inflammation than those without inflammation (*P* < 0.001; 5.13 ± 1.21) (Fig. 5C).

A significantly lower BAMBI mRNA expression level was found in patients with inflammatory status compared with patients without inflammatory infiltrate (Fig. 5D) (*P* < 0.001). Immunohistochemistry demonstrated that BAMBI was expressed in both glandular epithelium cells and some stromal cells in all BPH specimens. BAMBI staining was detected in the cytoplasm and several nuclei of glandular epithelium cells. There was a slightly higher BAMBI staining score in the non-inflammatory group, 4.25 ± 1.10, compared with 3.63 ± 1.27 in the inflammatory group, although this difference did not reach statistical significance (Fig. 5E) (*P* = 0.095).

The up-regulation of p-Smad2/3 in prostate epithelial cells indicates the activation of the TGF-β/Smad signalling pathway and is associated with EMT. BPH tissues without inflammatory infiltrate showed only a low level of p-Smad2/3 expression (staining grade = 3.37 ± 1.29) by immunohistochemistry. In these biopsy specimens, p-Smad2/3 was scattered in the nuclei of some prostate epithelial cells. In marked contrast, biopsy specimens from patients with inflammatory status showed strong endonuclear p-Smad2/3 expression (staining grade = 5.11 ± 1.48) in most prostate epithelial cells (Fig. 5F) (*P* < 0.001).

### Association between the expression of TLR4 mRNA and clinical findings in the inflammatory group

**Figure 6:**
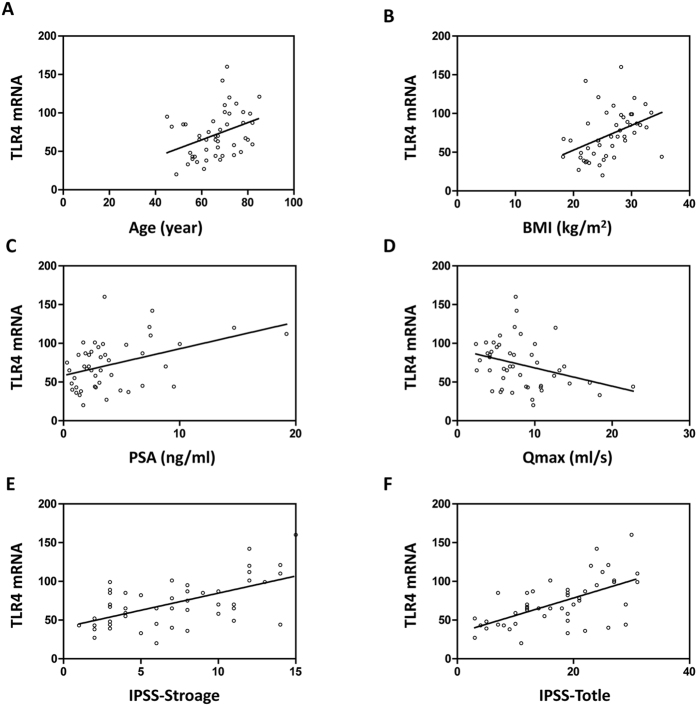
Correlation between TLR4 mRNA expression and clinical data. In the inflammatory group, TLR4 mRNA expression level is significantly correlated with age (**A**), BMI (**B**), serum PSA levels (**C**), Qmax (**D**), storage IPSS (**E**) and total IPSS (**F**).

We investigated the correlation between the level of TLR4 mRNA in the prostate of BPH patients with inflammatory infiltrate and clinical data such as age, BMI, PSA, IPSS, QOL index, PVR, or Qmax (Table 2). In overall 47 patients, TLR4 mRNA expression level was significantly positively associated with age (r^2^ = 0.153, *P* = 0.007), BMI (r^2^ = 0.382, *P* < 0.001) and serum PSA levels (r^2^ = 0.190, *P* = 0.002), and negatively associated with Qmax (r^2^ = −0.137, *P* = 0.011) (Fig. 6A–D). However, TLR4 mRNA expression level was not significantly associated with total prostate volume (TPV) or PVR.

Next, we correlated the expression TLR4 mRNA with each item subscore of IPSS using Pearson’s correlation coefficient analyses. There was a significant association between the level of TLR4 mRNA and urgency (r^2^ = 0.194, *P* = 0.005), nocturia (r^2^ = 0.186, *P* = 0.004). However, we observed no significant association between the level of TLR4 mRNA and frequency, incomplete emptying, intermittency, straining to void, weal stream or QOL index. TLR4 mRNA expression level was positively associated with total IPSS (r^2^ = 0.137, *P* = 0.011) and IPSS-S (r^2^ = 0.153, *P* = 0.007), but not with IPSS-V (Fig. 6E,F).

## Discussion

BPH is one of the most frequent chronic diseases in the aging male population. Its incidence gradually increases with age; more than two-thirds of men aged over 50 have histological evidence of BPH and about half of the male population older than 50 years suffers from BPH-associated LUTS symptoms, such as urgency, frequency and retention^25^,^26^. Aging and the presence of androgens are necessary for the development of BPH, but its pathogenesis still remains unresolved^2^,^3^. Accumulating evidence suggests that intraprostatic inflammation and inflammatory factors play a critical role in the pathogenesis of BPH and in the aggravation of its clinical symptoms^27^,^28^,^29^,^30^.

Epithelial cells are the first lines of host defence against invading bacteria and other pathogens. Prostate epithelial cells secrete inflammatory cytokines that lead to the activation of signalling pathways that regulate cell proliferation, apoptosis and differentiation^6^,^7^. TLR4, a family member of transmembrane receptors, plays an important role in signalling pathways of the innate immune response to infection by several pathogens^10^. TLR4 is expressed in macrophages, neutrophils and other immunity cells, as well as epithelial cells. Some studies demonstrated that TLR4-mediated pathways are involved in the process of chronic inflammation inducing organ fibrosis. However, little is known about the response of human prostatic epithelial cells to TLR4 signalling. In the present study, we found that LPS/TLR4 can enhance the EMT process in BPH-1 cells.

Pei *et al.*^20^ showed that human prostate epithelial PC3 cells constitutively express TLR4 mRNA and protein levels and the expression and secretion of TGF-β1, vascular endothelial growth factor and IL-6 were up-regulated post-LPS treatment. Their results indicated that TLR4 signalling may affect TGF-β1 signalling in prostatic epithelial cells and further promote development and progression of prostatic diseases. Gatti *et al.*^31^ found that rat prostate epithelial cells constitutively express high levels of TLR4, TLR2 and CD14 upon LPS treatment. They further elucidated that functional activation of TLR4 is essential for the induction of the expression of nitric oxide (NO) synthase and secretion of NO. Kim *et al.*^21^ observed profound EMT features in LPS-treated rat prostates.

Increasing evidence has suggested that EMT is associated with a number of pathological organ fibrosis, such as hepatic fibrosis, renal fibrosis, and peribiliary fibrosis^32^,^33^,^34^. TGF-β is a pleiotropic cytokine associated with the induction and maintenance of EMT in various normal and cancer cell types. Magdalena *et al.*^35^ analysed prostatic samples from 16 BPH patients by IHC and found that three key downstream elements in TGF-β signalling, Smad3, Snail, and Slug, were up-regulated in BPH tissue. Staršíchová *et al.*^19^ found that EMT was induced by TGF-β1 treatment in BPH-1 cells and showed that TGF-β1 at 10 ng/ml could drive EMT in BPH-1 cells, while 0.1 ng/ml TGF-β1 could not induce EMT and only slightly induced phosphorylation of Smad2.

In the present study, we used 10 ng/ml TGF-β1 as the positive control and 0.1 ng/ml TGF-β1 as the negative control, based on the literature, and our results were consistent with previous studies. Our results showed that LPS or 0.1 ng/ml TGF-β1 treatment alone had no significant effect on E-cadherin or vimentin expression, however LPS combined with 0.1 ng/ml TGF-β1 led to a reduction in E-cadherin expression and increase in vimentin levels. This finding showed that LPS enhanced the sensitivity of BPH-1 cells to the stimulatory effect of TGF-β1. We also showed that the synergistic effect of LPS and TGF-β1 (0.1 ng/ml) in upregulating vimentin expression and downregulating E-cadherin expression was abrogated by the TLR4 inhibitor CLI-095, demonstrating that LPS-mediated TLR4 activation plays a crucial role in augmenting the TGF-β dependent EMT response in BPH-1 cells. In contrast to our results, Kim *et al.*^36^ suggested that LPS stimulus inhibited rather than enhanced TGF-β/Smad signalling in RAW264.7 cells. This divergence of LPS responses might be explained by the cell specificity, with inflammatory cells showing an inhibitory cross talk between TLR4 and TGF-β, whereas a positive cross talk is observed in epithelial and mesenchymal cells.

In addition to the canonical TGF-β-Smad pathway, the biological effects of TGF-β1 can also be mediated by non-canonical TGF-β signalling pathways. Chen *et al.*^37^ showed that TGF-β1 induced EMT in A549 cells through non-Smad-dependent mechanisms following non-canonical PI3K/AKT and MAPK/ERK1/2 signalling pathway activation. Our results showed that the combination of LPS and TGF-β1 (0.1 ng/ml) induced a significant upregulation of p-Smad2/3 protein levels, which was completely abrogated by CLI-095 pretreatment. This suggests that TLR4 signalling in BPH-1 cells directly affects the canonical Smad pathway of TGF-β1 signalling.

The pseudoreceptor BAMBI is a negative regulator of the TGF-β signalling pathway that exhibits structural homology to TGF-βRI but lacks the intracellular kinase domain. Several studies have demonstrated that BAMBI is expressed in many human organs and is involved in organ fibrogenesis and cancer development. Pulskens *et al.*^38^ reported that reduced fibrosis in TLR4-deficient mice after unilateral ureteral obstruction might be explained by a decreased susceptibility of renal cells to TGF-β as a result of higher expression of BAMBI. Bhattacharyya *et al.*^39^ found that TLR4 was constitutively expressed in lesional skin and lung tissues and was related to extracellular matrix remodelling and fibrogenesis. The authors also suggested that TLR4-mediated BAMBI downregulation sensitises fibroblasts to TGF-β signalling, resulting in progressive fibrogenesis. This finding is consistent with the study of Seki *et al.*^40^ that suggested that BAMBI might be involved in LPS/TLR4-mediated hepatic fibrosis.

Based on these studies, we speculated that BAMBI might function as a critical link between LPS/TLR4 and TGF-β/Smad signalling pathways. We found that the downregulation of BAMBI after LPS treatment was abrogated by the TLR4 inhibitor CLI-095. These data indicate that activation of LPS/TLR4 axis might downregulate BAMBI mRNA and protein expression. Loss of BAMBI could enhance the intensity of Smad-mediated TGF-β1 responses. Therefore, the observed decrease in BAMBI expression following TLR4-dependent activation by LPS in these experiments might be, in part, responsible for the LPS/TLR4 axis augmenting the TGF-β1 signalling to induce EMT process in BPH-1 cells. Additionally, double immunofluorescent analysis revealed that TLR4 and BAMBI were colocalised in the cell membrane and cytosol, indicating that BAMBI is co-expressed with TLR4 in the same prostate epithelial cells.

Next we infected BPH-1 cells with adenoviruses expressing AdBambi or AdshBambi, and found that LPS in the presence of 0.1 ng/ml TGF-β1 did not induce vimentin and p-Smad2/3 expression or decrease E-cadherin expression in AdBambi-infected BPH-1 cells. These results provide evidence supporting a pivotal role of BAMBI in TLR4 augmenting the TGF-β1-dependent EMT response. Interestingly, there were no significant differences between the AdshBambi group and the control group after LPS (100 ng/ml) plus TGF-β1 (0.1 ng/ml) treatment. This might be explained by the overwhelming effect of the LPS. BAMBI expression was down-regulated by LPS in control group. While in AdshBambi group, BAMBI expression was affected by both AdshBambi transfection and LPS treatment. Thus, it is difficult to discern additional effects of AdshBambi transfection.

Acute and chronic prostate inflammation is typically detected in BPH specimens. Several studies have reported that inflammatory signs were found in 43–98% of specimens obtained from aging men^41^. In this study, we analysed samples obtained by TURP from 62 BPH patients and found that 47 patients (76%) showed inflammatory infiltration within the prostate tissue samples, which is consistent with previous studies. Moreover, we found that patients with inflammatory status were more likely to have a higher age (*P* = 0.020) and BMI (*P* = 0.026), and TLR4 mRNA expression level was significantly positively associated with age (r^2^ = 0.153, *P* = 0.007) and BMI (r^2^ = 0.382, *P* < 0.001). These results might indicate that elder and obese patients are more likely to have evidence of inflammation in prostatic specimens.

The Medical Therapy of Prostatic Symptoms (MTOPS) trial confirmed that patients with prostate inflammation were older, had a larger prostate volume, and a higher serum PSA level than patients without prostate inflammation^42^. Similarly, the Reduction by Dutasteride of Prostate Cancer Event (REDUCE) trial examined inflammation and 77.6% of the patients had chronic inflammation in prostatic biopsy specimens obtained from 8824 patients. Patients with inflammation had a larger prostate volume (46.5 vs. 43.4 ml; *P* < 0.0001) and higher IPSS score than patients without inflammation (8.8 vs. 8.2; *P* < 0.0001)^43^. Our results demonstrated that patients with inflammatory infiltrate were more likely to have larger prostate volume (*P* = 0.024), higher total IPSS score (*P* = 0.009) and IPSS-S (*P* < 0.001) than those without inflammation. In addition, we found that patients with inflammatory status had higher frequency (*P* = 0.008), urgency (*P* = 0.011) and nocturia (*P* = 0.036) subscores of IPSS. Taken together, these studies indicated that inflammation might contribute to the development and progression of BPH and LUTS.

Multiple studies have shown that TLR4 is expressed in mucosal epithelia, such as in intestine^44^, respiratory tract^45^, oral cavity^46^, and urinary tract^7^. Despite the high incidence of BPH, little is known about TLR4 expression in the human prostate tissues. Our study demonstrated that TLR4 is mainly located in the cytoplasm of luminal epithelium cells and that TLR4 mRNA expression level was higher in BPH tissues with inflammatory status. TLR4 plays a pivotal role in the process of chronic inflammation inducing BPH, and examining TLR4 expression levels might contribute to the diagnosis of BPH with inflammatory infiltrate.

According to previous reports, storage symptoms were known as the most prevalent and bothersome symptoms in patients with symptomatic BPH^47^. The results of this study demonstrated that the TLR4 mRNA expression level in BPH tissues with inflammatory status increased with age, higher BMI, higher PSA levels, and smaller Qmax. Moreover, we found that TLR4 mRNA expression level was positively associated with total IPSS (r^2^ = 0.137, *P* = 0.011) and IPSS-S (r^2^ = 0.153, *P* = 0.007), but not with IPSS-V. These results suggested that inflammation might play a role in the BPH patients with storage predominant LUTS.

In conclusion, modulation of TGF-β induced EMT by LPS/TLR4 axis provides a link between prostatic hyperplasia and inflammation. Targeting TLR4 mediated signalling may represent a novel therapeutic approach for the treatment of BPH.

## Materials and Methods

### Cell culture and stimulation

BPH-1 cells were obtained from the Shanghai Jiao Tong University and Cell Cultures. The cells were cultivated in RPMI 1640 supplemented with 20% bovine foetal serum, 5 μg/ml transferrin, 5 ng/ml sodium selenite, 5 μg/ml insulin, streptomycin (0.1 mg/ml), and penicillin (100 U/ml). Tumorigenic clones of BPH-1 cells were a generous gift from Dr Shu-Jie Xia (Shanghai Jiao Tong University). The cell lines were cultivated in a humidified incubator at 37 °C in 5% CO_2_.

For experiments, BPH-1 cells were cultured in serum-free media containing 0.1% bovine serum albumin for 24 h before treatment with highly purified LPS (Sigma, St. Louis, MO, USA) and various concentrations of TGF-β1 (PeproTech, Rocky Hill, NJ, USA). In selected experiments, BPH-1 cells were incubated with the selective TLR4 inhibitor, CLI-095 (InvivoGen, San Diego, CA, USA) for 30 min before the addition of LPS or TGF-β1.

### Plasmid Constructs and Viruses Recombinations

The shRNA for target BAMBI mRNA was subcloned into the Hind III and Bam HI restriction sites of pGenesil-1 (Wuhan GeneSil Biotechnology, China). The DNA sequences coding the shRNA for BAMBI are reported before^48^. Human BAMBI cDNA was purchased from OriGene Technologies and cloned into pENTR vector. The pGenesil-1 vector or BAMBI-pENTR was homologously recombined with pAd/PL-DEST vector into DH5α bacteria. Ampicillin and Chloramphenicol LB plates were applied for the selection. The complete virus was recovered by transfection of 10 mg Pac I digested DNA into human HEK 293 cells by using lipofectamine-based procedure.

### Patients and samples

Between June 2012 and December 2013, 62 BPH prostate specimens obtained by transurethral resection of the prostate (TURP) were collected at our institution. TURP was performed owing to lower urinary tract symptoms (LUTS) associated with obstructive benign prostatic disease. Patients with prostate cancer were excluded from this study. We used bipolar TURP, working in isotonic saline solution. The specimens were immediately frozen after TURP and stored at −80 °C in the Xiangya Hospital, Changsha, China. All specimens were used for haematoxylin and eosin (H&E) staining to determine grouping according to presence of inflammatory infiltrate. The mean age in the inflammatory group and non-inflammatory group was 65.9 ± 8.12 and 60.3 ± 7.04 years old, respectively. Informed written consent was obtained from all patients. The usage of patient material was approved by the ethics committee at Xiangya Hospital of Central South University, and in accordance with the guidelines of Xiangya Hospital of Central South University.

### RNA isolation and real-time RT-PCR

Total RNAs were extracted from BPH-1 cells or frozen tissue specimens with Trizol reagent (Invitrogen, Carlsbad, CA, USA) according to the manufacturer’s protocol. Total RNA concentration and protein contamination were determined by a spectrophotometer (BioPhotometer, Eppendorf, Germany) and further verified on a 2100 Bioanalyzer (Agilent, Santa Clara, CA, USA), and stored at −80 °C. RNA integrity was verified by visualising 18S and 28 S RNA bands under a UV light. The Nanodrop A260:280 ratio of the samples were between 1.8 and 2.0.

RNA was converted to cDNA using specific primers. TLR4, BAMBI, E-cadherin, and vimentin mRNA expression was analysed by real-time quantitative RT-PCR performed on an AB cycler with SYBR Green PCR Master Mix (Applied Biosystems, Foster City, CA). The primers are commercially available from Applied Biosystems as assays-on-demand. The results are expressed as fold changes in comparison with untreated groups and normalised with GAPDH.

### Western blot

BPH-1 cells were lysed, and total protein was harvested and quantitated using standard Bradford assays. Protein samples were separated by SDS-polyacrylamide gel electrophoresis and then transferred to nitrocellulose membranes. Expression of the E-cadherin, vimentin, p-Smad2/3, and BAMBI was monitored by the enhanced chemiluminescence western blotting system (Pierce, Rockford, IL, USA) according to the manufacturer’s protocol.

### Fluorescent immunocytochemistry

BPH-1 cells were fixed in 4% formaldehyde/PBS for 30 min at room temperature and permeabilised with 0.5% Triton X-100/PBS for 20 min after washing with PBS. Cells were incubated with 3% H_2_O_2_/PBS for 20 min at room temperature to block endogenous peroxidase activity. Subsequently, cells were blocked for 20 min with 0.2% fish gelatine/PBS and incubated with primary antibodies against TLR4 (1:50; Santa Cruz Biotechnology Inc., M-16), BAMBI (1:50; Bioss., bs-12418 R) overnight at 4 °C. After washing, biotinylated secondary antibodies were added and incubated for 60 min at room temperature. Cell nuclei were counterstained with DAPI (1 μg/ml; Roche). The stained cells were examined under a fluorescence microscope (Olympus, Tokyo, Japan).

### Immunohistochemistry and immunocytochemistry

For immunocytochemical analysis, we fixed BPH-1 cells in 4% formaldehyde/PBS for 30 min at room temperature and permeabilised cells with 0.5% Triton X-100/PBS for 20 min after washing with PBS. Cells were incubated with 3% H_2_O_2_/PBS for 20 min at room temperature to block endogenous peroxidase activity. Subsequently, cells were blocked for 20 min with 0.2% fish gelatine/PBS and incubated with primary antibodies against E-cadherin (1:200; Abcam, ab1416), vimentin (1:200; Abcam, ab92547), p-Smad2/3 (1:50; Santa Cruz Biotechnology Inc., sc-11769), GFP (1:500; Rockland, 600-101-215), TLR4 (1:50; Santa Cruz Biotechnology Inc., M-16), or BAMBI (1:50; Bioss., bs-12418 R) overnight at 4 °C. After washing, biotinylated secondary antibodies were applied for 30 min followed by incubation with a horseradish peroxidase-streptavidin detection system (Dako) for 20 min according to the manufacturer’s instructions.

For immunohistochemical staining, formalin-fixed, paraffin-embedded tissues from the 62 BPH prostate specimens were cut in 5-μm-thick sections, dewaxed, and rehydrated. Antigen retrieval was performed using 10 mM citrate buffer (pH 6.0) in a microwave oven. Endogenous peroxidase activity was blocked by incubation for 20 min with 3% H_2_O_2_/PBS. Sections were incubated with primary antibodies against p-Smad2/3 (1:50; Santa Cruz Biotechnology Inc., sc-11769), GFP (1:500; Rockland, 600-101-215), TLR4 (1:50; Santa Cruz Biotechnology Inc., M-16), or BAMBI (1:50; Bioss., bs-12418R) for 1 h at room temperature. After washing with PBS, sections were incubated with the corresponding secondary antibodies for 20 min at room temperature, followed by incubation with a horseradish peroxidase-streptavidin detection system (Dako) for 20 min according to the manufacturer’s instruction. The visualisations were done using a light microscope.

### Evaluation of staining

All sections were evaluated by two separate pathologists (D. Z. H. and L. X.) in a blinded manner under a transmission light microscope. In cases of occasional scoring discrepancy, consensus was always achieved after discussion of findings. For TLR4, BAMBI and p-Smad2/3 assessment, the entire tissue section was scanned to assign the scores. Intensity of staining was scored as 0 (negative), 1 (weak), 2 (medium), or 3 (strong). The staining extent was scored as 0 (0%), 1 (1–25%), 2 (26–50%), 3 (51–75%), and 4 (76–100%), according to the percentage of positive staining areas in relation to the whole prostatic hyperplasia area. The sum of the extent and intensity score was used as the final staining score (0–7).

### Statistical analysis

All experiments were repeated at least three times. Data are presented as mean with standard deviation. Multifactorial ANOVA was used for comparisons between experimental and control groups. Patients were subdivided into two groups: those with or without prostate inflammation. Differences between groups were analysed using Wilcoxon rank sum test. Pearson’s correlation coefficient and multiple regression analyses tested the association between TLR4 mRNA expression level as the dependent variables and patient age, BMI, IPSS-total, IPSS-V, IPSS-S, individual symptoms of IPSS, QOL score, total PSA, TPV, Qmax or PVR as the independent variables. A *P* value less than 0.05 was considered statistically significant (two-tailed). SPSS version 18.0 statistical software (SPSS Inc., Chicago, IL, USA) was used for analysis of all data.

## Additional Information

**How to cite this article**: He, Y. *et al.* LPS/TLR4 Signaling Enhances TGF-ß Response Through Downregulating BAMBI During Prostatic Hyperplasia. *Sci. Rep.* **6**, 27051; doi: 10.1038/srep27051 (2016).

## Figures and Tables

**Table 1:** Patient characteristics and descriptive statistics.

Variables	Patient Inflammation		P Value
	With	Without	
Patients	47	15	
Age (y)	65.9 ± 8.12	60.3 ± 7.04	0.020
BMI (kg/m2)	28.4 ± 5.03	25.1 ± 4.27	0.026
Total PSA (ng/ml)	5.25 ± 3.22	4.71 ± 1.92	0.542
Prostate volume (ml)	58.3 ± 27.9	40.7 ± 16.2	0.024
IPSS-Total	17.2 ± 6.12	12.5 ± 5.16	0.009
Incomplete emptying	1.85 ± 1.63	1.27 ± 1.05	0.202
Frequency	3.46 ± 2.35	1.71 ± 1.26	0.008
Intermittency	2.37 ± 2.65	3.05 ± 2.69	0.392
Urgency	2.47 ± 2.01	1.03 ± 1.16	0.011
Weak stream	2.78 ± 2.61	3.15 ± 3.03	0.647
Straining	1.26 ± 1.83	0.97 ± 1.14	0.464
Nocturia	2.97 ± 2.84	1.32 ± 1.56	0.036
IPSS-V	8.26 ± 4.45	8.44 ± 4.47	0.892
IPSS-S	8.90 ± 4.20	4.06 ± 2.57	< 0.001
QOL score	4.18 ± 1.95	3.52 ± 1.87	0.254
Qmax (ml/s)	6.37 ± 4.74	7.48 ± 2.13	0.385
PVR (ml)	19.4 ± 8.72	22.3 ± 7.15	0.237
TLR4 mRNA expression level	4.92 ± 1.15	1.03 ± 0.27	< 0.001
BAMBI mRNA expression level	0.46 ± 0.17	1.05 ± 0.22	< 0.001
TLR4 staining score	5.13 ± 1.21	2.96 ± 0.73	< 0.001
BAMBI staining score	3.63 ± 1.27	4.25 ± 1.10	0.095
p-Smad2/3 staining score	5.11 ± 1.48	3.37 ± 1.29	< 0.001

BMI, body mass index; PSA, prostate specific antigen; IPSS, International Prostate Symptom Score; V, voiding subscore; S, storage subscore; QOL, quality of life; Qmax, maximum flow rate; PVR, postvoid residual.

**Table 2:** Correlation between the expression TLR4 mRNA and clinical data.

Variable	Pearson correlation coefficient	p value
Age	0.153	0.007
BMI	0.382	< 0.001
IPSS-Total	0.137	0.011
Incomplete emptying	0.037	0.398
Frequency	0.051	0.441
Intermittency	0.004	0.903
Urgency	0.194	0.005
Weak stream	−0.009	0.852
Straining	−0.017	0.526
Nocturia	0.186	0.004
IPSS-V	0.041	0.297
IPSS-S	0.153	0.007
QOL score	0.065	0.384
Total PSA (ng/ml)	0.190	0.002
TPV (ml)	−0.012	0.733
Qmax (ml/s)	−0.137	0.011
PVR (ml)	0.048	0.457

BMI, body mass index; PSA, prostate specific antigen; IPSS, International Prostate Symptom Score; V, voiding subscore; S, storage subscore; QOL, quality of life; TPV, total prostate volume; Qmax, maximum flow rate; PVR, postvoid residual.
